# Asthaxanthin Improves Aerobic Exercise Recovery Without Affecting Heat Tolerance in Humans

**DOI:** 10.3389/fspor.2019.00017

**Published:** 2019-09-04

**Authors:** Chen Fleischmann, Michal Horowitz, Ran Yanovich, Hany Raz, Yuval Heled

**Affiliations:** ^1^Institute of Military Physiology, IDF Medical Corps, Tel-Hashomer, Israel; ^2^Heller Institute of Medical Research, Sheba Medical Center, Ramat Gan, Israel; ^3^Laboratory of Environmental Physiology, Dentistry Faculty, Hebrew University of Jerusalem, Jerusalem, Israel; ^4^The Academic College at Wingate, Wingate Institute, Netanya, Israel; ^5^The Faculty of Agriculture, Food and Environment, Hebrew University, Rechovot, Israel

**Keywords:** astaxanthin, supplementation, exercise nutritional physiology, aerobic exercise, exercise-recovery, heat tolerance

## Abstract

**Objectives:** To examine the supplementation effects of the xanthophyll carotenoid Astaxanthin on physical performance and exertional heat strain in humans.

**Design:** A randomized double blind placebo controlled trial.

**Methods:** Twenty two male participants (Age: 23.14 ± 3.5 y, height: 175 ± 6 cm, body mass: 69.6 ± 8.7 kg, % body fat: 16.8 ± 3.8) received placebo (PLA, *n* = 10) or Astaxanthin (ATX, *n* = 12) 12 mg/day Per os (P.O), for 30 days, and were tested pre and post-supplementation with a maximal oxygen uptake (VO_2_ Max) test and the heat tolerance test (HTT) (2 h walk at 40°C, 40% relative humidity (RH), 5 kph, 2% incline). NIH database registration no. NCT02088242. Gas exchange, Heart rate (HR), Relative perceived exertion (RPE), and blood lactate were measured during the VO_2_ Max test. Heart rate (HR), rectal (Trec), and skin (Tskin) temperatures, RPE, and sweat rate (SR) were monitored in the HTT. Serum heat shock protein 72 (HSP72), Creatine phospho-kinase (CPK), C-reactive protein (CRP), and lipid profile were measured before and after the test.

**Results:** The rise in blood lactate caused by the VO_2_ Max test was significantly diminished in the ATX group (9.4 ± 3.1 and 13.0 ± 3.1 mmole^*^l^−1^ in the ATX and PLA groups, respectively *P* < 0.02), as was the change in oxygen uptake during recovery (−2.02 ± 0.64 and 0.83 ± 0.79% of VO_2_ Max in the ATX and PLA group, respectively, *p* = 0.001). No significant differences were observed in the anaerobic threshold or VO_2_ Max. In the HTT, no significant physiological or biochemical differences were observed (HR <120 bpm, Trec rose by ~1°C to <38°C, no difference in SR).

**Conclusions:** Astaxanthin supplementation improved exercise recovery. No benefit was observed for ATX over PLA in response to heat stress. Further examination of Astaxanthin in higher exertional heat strain is required.

## Introduction

Astaxanthin is a xanthophyll carotenoid food supplement prevalent in marine organisms (Kidd, 2011). This potent antioxidant (Kidd, 2011) affects the Insulin\Insulin growth factor I (IGF1) and the nuclear kinase mitogen and stress-activated protein kinase-1 (MSK1) signaling pathways, which were found to be implicated in preconditioning, survival and longevity *in vitro* in human keratinocytes (Terazawa et al., 2012) and *in vivo* in *Caenorhabditis elegans* (Yazaki et al., 2011) Other *in vivo* experiments in animals have shown Astaxanthin is associated with reductions in C reactive protein and DNA damage and improvement of the cell-mediated and humoral immune responses (Park et al., 2011) and with improvement in cardiovascular parameters (Fassett and Coombes, 2012). In exercising mice Astaxanthin induced diminished fatigue, and reductions in blood lactate, oxidative damage to lipids and DNA, and muscle injury (Aoi et al., 2003; Ikeuchi et al., 2006). However, exercise experiments in humans were equivocal, showing improved endurance as time trial performance in competitive cyclists (Earnest et al., 2011), vs. no significant improvement in well-trained cyclists (Res et al., 2013) and soccer players (Djordjevic et al., 2012). During exercise, some evidence from animal experiments supports enhanced fat utilization over carbohydrates (Ikeuchi et al., 2006; Aoi et al., 2008), yet no supplementation effect in endurance exercise and recovery was established (Brown et al., 2018).

Exertional heat injury is a life threatening condition inflicting many young, healthy individuals, commonly affecting highly motivated, physically active populations such as military personnel and athletes (Carter et al., 2005; Casa et al., 2005). Risk reduction of heat injury includes avoidance of strenuous physical activity in severe heat load conditions, application of heat acclimation protocols, and the use of external cooling methods (Epstein et al., 2000). Preparation for planned military and athletic activities could potentially enhance resilience to extreme physical and environmental conditions, reduce the chance of heat injury and improve injury response and recovery. Several exogenous agents have been studied either as prophylactic to heat stress exposure or as post injury treatment yet none were effective (Moran et al., 1999; Kuennen et al., 2011).

Heat load is a significant stressor during exercise. Astaxanthin's activity against stressor induced generation of reactive oxygen and nitrogen species (RONS) and inflammatory cytokines (Brown et al., 2018) may thus be beneficial in heat stress conditions. Accordingly, Do et al. demonstrated that during development, treatment with Astaxanthin increased protection of porcine oocytes against heat shock, along with increased resilience to oxidative stress (Do et al., 2015). In rodents, Astaxanthin enhanced protection against heat related damages and oxidative stress (Preuss et al., 2009) and resilience against heat stress combined with gravitational unloading (Yoshihara et al., 2018). In a preliminary experiment our group demonstrated improved heat tolerance with elevated cardiac tissue concentration of HSP72 protein and HSP70 mRNA in rats (Horowitz M. & Abbas A., unpublished data). In yellow catfish, Astaxanthin pretreatment improved overall stress resistance, while elevating hepatic heat shock protein 70 (HSP70) mRNA levels, with increased antioxidant capacity, and decreased expression of the stress related hormone cortisol and glucose levels (Liu et al., 2016). Pufferfish fed with a diet containing Astaxanthin produced less reactive oxygen species (ROS) when exposed to heat stress, and increased production of superoxide dismutase (SOD), catalase (CAT), and HSP70 mRNA under high temperature stress, in comparison with the control (Cheng et al., 2018). Elevation of HSP70 mRNA and HSP72 protein is an important part of the heat shock response (HSR), representing innate cellular defense mechanisms against heat related damage (Horowitz, 1998). Overall, across several animal models, Astaxanthin treatment enhances cellular protection against heat with corresponding increased levels of HSP70, possibly by priming key components of the HSR for activation, and by acting as a potent antioxidant via protection against the heat stress induced generation of RONS.

Based on the aforementioned knowledge, and in the absence of known safe substances applicable as preemptive measures for anticipated heat stress exposure, Astaxanthin emerges as a potential candidate for enhancing heat resilience through increased cellular protection, pertinent to both heat and exercise exposure, as it is a safe food supplement, which may potentially be consumed chronically without adversely affecting active populations. Therefore, we set out to determine whether Astaxanthin supplementation, as a preemptive strategy, could have an influence on performance in heat stress combined with exercise scenarios in humans, and potentially serve as an added line of defense against heat related injury for individuals anticipating exposure to heat and exercise. We also chose to separately evaluate the influence of Astaxanthin supplementation on aerobic fitness, since it is a key contributing component to endurance in the heat (Mclellan et al., 2012) and to determine whether the potential added cellular protection might influence aerobic performance, independently of heat exposure.

The study goals were to determine whether Astaxanthin pre-supplementation could influence performance in exercise alone or in combination with heat stress.

## Methods

In order to evaluate the influence of Astaxanthin supplementation on heat tolerance and on aerobic capacity, we employed a double blind placebo controlled randomized trial. The heat tolerance test (HTT), which involves exposure to mild physical activity in controlled heat load conditions and the maximal oxygen uptake (VO_2_ Max) test were used before supplementation and repeated after 1 month of daily supplementation. The study was approved by the ethical review boards of the Sheba medical center (reg. no. 1295-13) and of the IDF Medical Corps (reg. no. 0471-13) and was registered in the NIH database (reg. no. NCT02088242). Data collection took place between March of 2015 and March of 2016 at the Heller institute of medical research located in the Sheba medical center, Tel-Hashomer, Israel.

### Participants

Twenty two young healthy male volunteers, free from illness and not consuming medications or dietary supplements, completed their participation in the study after giving their informed consent and being examined by the study physician. Participants were interviewed by a nutritionist to ensure an Astaxanthin free diet and instructed to avoid changing their exercise routine for the duration of the study, and refrain from consuming Astaxanthin containing foods, as well as any dietary supplements for 2 weeks prior to participating in the physical tests and throughout the duration of the study. They were randomly assigned to either the supplementation group, who received 12 mg of Astaxanthin P.O daily as 3 soft gel capsules of Astapure® (10% Oleoresin) 4 mg or a placebo identical in appearance and taste, which contained no Astaxanthin (Algatech, Ktora, Israel). The Supplement and placebo capsules were purchased directly from the manufacturer, to guarantee production of a placebo identical in every way to the supplement, apart from the presence of the active ingredient. Certificates of analysis were issued for each purchased batch, ensuring a 95% purity at least of the active ingredient (Astaxanthin) in the oleoresin contained in the soft gel capsules.

### Treatment

The dose (12 mg) was chosen in accordance with the highest daily dose approved for human consumption by the U.S. Food and Drug Administration (FDA) at the time of study approval and with literature evidence from human experimentation, demonstrating safety and efficacy at this and higher doses (Kupcinskas et al., 2008; Yoshida et al., 2010; Choi et al., 2011; Nakagawa et al., 2011). Supplementation duration (over 30 days) was chosen in order to ensure adequate time for achieving a supplemented state and initiating the necessary long term effects, based on other human experiments involving exercise related aspects, without any known threat to the subjects' health and well-being (Spiller and Dewell, 2003; Bloomer et al., 2005; Earnest et al., 2011; Miyazawa et al., 2011).

Randomization and assignment to the Astaxanthin or placebo group was performed by an independent party (the clinical research division of the Sheba medical center pharmaceutical services), which also individually dispensed the study product to the participants. Treatment allocation was disclosed to the researchers only after study completion. In order to ensure maximal gastro-intestinal (GI) absorption, participants were instructed to ingest the supplement or placebo with a meal containing 15 grams of fat. Supplementation lasted for 30 days, immediately followed by an additional supplementation period of 5–10 days, during which the physical tests (HTT and VO_2_ Max) were repeated, on separate days, in-order to maintain an effective concentration of the supplement and ensure the tests were performed under a supplemented state. Treatment adherence by participants was monitored by keeping a supplementation log and sending a daily text message after supplement consumption. A dietary log was also kept for 3 days before each physical examination day.

### Experimental Design and Procedures

**Figure 1:**
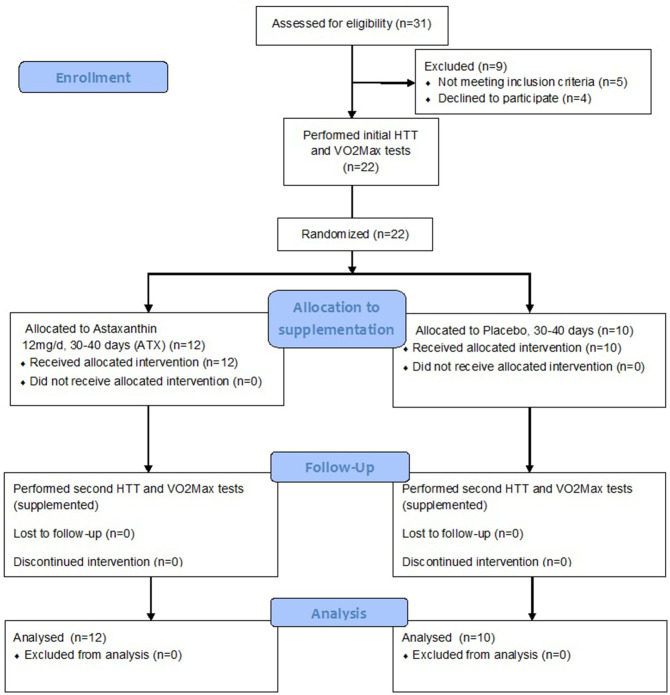
Study flow diagram.

Twenty two participants completed the study, after being randomly assigned to either the Astaxanthin (ATX, *n* = 12, age: 22.3 ± 4.0 years) or placebo (PLA, *n* = 10, age: 24.1 ± 2.60) groups in a double blind manner. Participants in both groups were of average anthropometrics (Height = 173.95 ± 4.0 cm, and 1.75 ± 7.6 cm; Body mass = 68.46 ± 8.0, and 70.96 ± 9.8; BMI = 22.6 ± 2.33, and 23.02 ± 2.40; %body fat = 13.32 ± 4.15% vs. 17.33 ± 3.41%, in the ATX and PLA groups, respectively, no significant difference between treatment groups). Supplementation began after completion of the initial HTT and VO_2_ Max tests, and lasted a total of 35–40 days. The HTT and VO_2_ Max tests where repeated after 30 days under ongoing supplementation. Figure 1 is a flow diagram of the study, detailing the process of participant recruitment, assignment and testing.

Anthropometric measurement (height, body mass, body fat from a four points skinfold measurement) was followed by evaluation of aerobic capacity and heat tolerance which were conducted on separate days, at least 48 h apart, and followed by commencement of daily supplementation. Aerobic capacity and heat tolerance assessment were repeated during the 31–40 day period of supplementation, while still consuming the supplement or placebo.

Anthropometry included height (roll-up stadiometer, model 206, Seca medical measuring systems and scales, Germany), body mass (electronic scales), and determination of body composition by the four sites (biceps brachii, triceps brachii, suprailiac, subscapular) skinfold measurement (Lange skinfold caliper, Beta technology, Santa Cruz, CA) and calculation of fat content and lean body mass, based on an equation suited to the participant's age (Durnin and Womersley, 1974).

The heat tolerance test (HTT) was described by Moran et al. (2007). Participants were dressed in shorts and tennis shoes and exposed to 2 h of extreme heat stress (40°C, 40% RH) in a climatic chamber, while walking on a motor-driven treadmill (5 kph, 2% incline). Rectal temp. (Trec), skin temp. (Tsk), and heart rate (HR) were continuously monitored. Fluid consumption (cold water) was provided ad-libitum from pre-weighed drinking cans. Trec was measured with a rectal thermistor (YSI-401, Yellow Springs Incorporated, USA) inserted 10 cm beyond the anal sphincter. Skin temp. (Tsk) at the chest, upper arm and calf, was measured using a skin thermistor (YSI-409B, Yellow Springs Incorporated, USA). Mean Tsk was calculated by Burton's equation (Burton, 1935). All temperatures were continuously recorded (MP150 and Acqknowledge software, version 3.9, Biopac systems, USA). Heart rate (HR) was continuously monitored by a heart rate monitor (model: RS800CX, POLAR, Finland). Blood pressure (BP) was monitored at pre-set time points (before the test, after 1 h of walking, at the end of the test, and every 15 min during recovery, for 1 h after the test) using an automated blood pressure monitor (Omron m6 comfort, Omron healthcare, Japan). Fluid balance was determined from nude body mass, measured before and after each trial, adjusted for fluid intake and urine volume, and used to calculate sweat loss, which was then normalized to body surface area and presented as the hourly sweat rate (SR). Relative perceived exertion (RPE) was assessed every 15 min during the HTT using the Borg scale (Borg, 1998), and a scale from 1 to 13 (unbearably cold to unbearably hot sensation, respectively), was used to rate the subjective sensation of thermal comfort (Thermal comfort rate, TCR). Safety thresholds for test cessation were set at Trec = 39°C or HR = 180 bpm, at the study physician's discretion or at the participant's request.

Maximal oxygen uptake (VO_2_ Max) was determined by cardio pulmonary exercise testing (CPET), using a modified Bruce protocol composed of 5 min seated rest, followed by 5 min of walking on a treadmill at 5 kph, and 2% incline, followed by running at 9 kph, with an incrementally increasing incline (2% every 2 min), until reaching VO_2_ Max, which was determined by 3 of 4 criteria during the test: (1) leveling off of the VO2 curve to a plateau, (2) reaching >90% of the participant's predicted maximal heart rate (210–0.65 × Age), (3) reaching a respiratory exchange ratio (RER) ≥1.1 or 4) at the participant's request, after reaching a subjective state of extreme physical tiredness. Additional supportive indications after test completion were reaching >8 mmol/l of blood lactate, or RPE > 17 (Edvardsen et al., 2014; Debeaumont et al., 2016). The ventilatory anaerobic threshold (AT) was determined visually by two trained examiners according to the American heart association guidelines (Balady et al., 2010). Continuous monitoring lasted throughout recovery, which consisted of 3 min at 5 kph and 2% incline, followed by 3 kph at 0% incline, and finally, 1 min, seated. The test was performed on a CPET machine (ZAN 600, Nspire Health, USA) connected to a treadmill ergometer (Model 770 S, RAM medical and industrial instruments, Germany). Reaching onset of blood lactate accumulation (OBLA) was confirmed by examining blood lactate level before and after the test (lactate scout+ analyzer, Sports Resource Group Inc., USA). Heart rate was continuously monitored by a heart rate monitor (model: RS800CX, POLAR, Finland). Assessment of RPE took place before and after the VO_2_ Max test.

Blood was drawn on physical testing days before the VO_2_ Max test and on HTT days before, immediately after and at 60 min after the end of the HTT. Blood was collected in yellow gel chemistry collection tubes (Becton, Dickinson and Co., NJ, USA), allowed to clot for 30 min and centrifuged. Serum was separated immediately and stored at −80°C pending analysis. Serum lipid and triglyceride (TG) profile, CRP, and CPK were analyzed by the central laboratories at the Sheba medical center. A commercially available ELISA kit for High-Sensitivity HSP72 detection was used to measure serum HSP72 levels in optical density (OD), which was used to calculate the HSP72 concentration in ng/ml, according to the manufacturer's instructions (AMP'D® HSP70 high sensitivity ELISA kit, ENZ-KIT-101, Enzo life sciences, NY, USA).

## Statistics

Anthropometric, physiological and biochemical parameters were statistically analyzed using the SPSS software (version 23, IBM, USA). Treatments and time point were taken as the independent variable and participants were considered a random sample of the general population. Normality of distribution was assessed by the Kolomogorov-Smirnov test and comparison between treatment groups and between pre- and post-supplementation time points was made with 1-way ANOVA, with Tukey *post hoc* analysis for normally distributing variables, or Mann-Whitney *U*-test for non-normally distributing variables. Analysis of the difference in the change in parameters due to supplementation between treatment groups was conducted by calculating the delta between the pre- and post-supplementation states (pre-supplemented state subtracted from the post-supplemented state). Normality of distribution was assessed by the Kolomogorov-Smirnov test and comparison between treatment groups was made by *T*-test for normally distributing variables and Mann-Whitney *U*-test for non-normally distributing variables. Leven's test was used to evaluate the equality of variance between treatment groups, followed by the appropriate Student's *t*-test (2-tailed) to assess significance. In order to assess the significance of difference between repetitive blood tests, ANOVA for repeated measures followed by Bonferroni *post-hoc* analysis or Friedman's omnibus test followed by Wilcoxon's signed-rank test with Bonferroni adjustment were used, for normally or non-normally distributing variables, respectively. A significant *p*-value was set at 0.05.

## Results

Table 1 lists key parameters of aerobic capacity, as recorded by the VO_2_ Max test. In both groups, anaerobic threshold (AT) was achieved at approximately 72% of the VO_2_ Max value, VO_2_ Max was similar and did not improve post-supplementation. Aerobic characteristics did not differ between the ATX and PLA groups both before and after supplementation, as seen in the unchanged AT, maximal oxygen uptake, reduction in heart rate during recovery, and in substrate utilization demonstrated by the scatter plot of respiratory exchange ratio (RER) vs. oxygen uptake (VO_2_) (Supplemental Figure 1).

**Table 1:** Main VO_2_ Max findings: This table lists the main findings from the maximal oxygen uptake tests performed before (pre) and after (post) supplementation in the two study groups.

**Test parameter**	**ATX pre**	**ATX post**	**PLA pre**	**PLA post**	**ANOVA/Mann-Whitney U**		**Delta ATX**	**Delta PLA**	***T*-test/Mann-Whitney U**
	**Mean ± St. Error**	**Mean ± St. Error**	**Mean ± St. Error**	**Mean ± St. Error**	***p*-value**	***Post hoc* Tukey *p*-value**	**Mean ± St. Error**	**Mean ± St. Error**	***p*-value**
RPE Before	6 ± 0	7 ± 0	7 ± 1	7 ± 0	N.S		0 ± 1	−2 ± 1	N.S
RPE After	18 ± 1	17 ± 0	16 ± 1	17 ± 0	N.S		−2 ± 2	−3 ± 3	N.S
Δ RPE	12 ± 1	11 ± 0	9 ± 1	11 ± 1	N.S		−2 ± 1	−1 ± 2	N.S
BLA before (mmole.l^−1^)	2.3 ± 0.31	2.57 ± 0.21	2.04 ± 0.24	2.08 ± 0.2	N.S		0.27 ± 0.37	−0.17 ± 0.38	N.S
BLA after (mmole.l^−1^)	13.46 ± 0.94	11.92 ± 0.85	12.65 ± 0.68	15.09 ± 1.02	N.S		−1.54 ± 0.85	0.93 ± 1.84	N.S
Δ BLA (mmole.l^−1^)	11.16 ± 0.78	9.35 ± 0.89	10.61 ± 0.73	13.01 ± 1.05	0.044	0.027*	−1.81 ± 0.89	1.1 ± 1.67	N.S
Sys. BP before (mmHg)	119 ± 2.77	120.64 ± 3.96	118.5 ± 2.87	121.29 ± 2.9	N.S		−8.42 ± 10.34	−33.6 ± 19.81	N.S
Dias. BP before (mmHg)	78.58 ± 3.14	77.64 ± 3.65	72.7 ± 3.09	79.57 ± 5.07	N.S		−7.42 ± 8.94	−17 ± 14.49	N.S
Sys. BP after (mmHg)	118.9 ± 4.42	127.2 ± 3.84	122.9 ± 5.34	117.71 ± 5.51	N.S		6.92 ± 21.73	−40.5 ± 17.89	N.S
Dias. BP after (mmHg)	75.1 ± 2.93	79.4 ± 3.11	76 ± 3.24	73.29 ± 4.36	N.S		3.58 ± 14.96	−24.7 ± 11.12	N.S
AT VO_2_ (ml.kg^−1.^min^−1^)	37.51 ± 3.35	40.1 ± 1.97	37.27 ± 3.51	38.53 ± 3.98	N.S		2.59 ± 3.68	1.26 ± 2.09	N.S
AT (% of VO_2_ Max)	72.32 ± 6.08	78.27 ± 3.17	71.3 ± 3.77	73.23 ± 4.51	N.S		5.95 ± 6.86	1.94 ± 3.79	N.S
Max load (Watt)	284.67 ± 11.46	288.5 ± 12.66	276.7 ± 19.6	281.1 ± 19.06	N.S		3.83 ± 5.92	4.4 ± 2.61	N.S
VO_2_ Max (ml.kg^−1.^min^−1^)	52.55 ± 1.83	51.24 ± 1.6	51.49 ± 2.32	51.54 ± 2.66	N.S		−1.31 ± 0.7	0.05 ± 0.71	N.S
HR Max (bpm)	190.67 ± 2.85	191.17 ± 2.33	191.2 ± 2.62	190.1 ± 2.85	N.S		0.5 ± 1.58	−1.1 ± 1.42	N.S
RER Max	1.14 ± 0.02	1.13 ± 0.02	1.15 ± 0.02	1.15 ± 0.03	N.S		0 ± 0.02	0 ± 0.02	N.S
^#^End recov. VO_2_ (ml*kg^−1^*min^−1^)	12.08 ± 0.32	10.76 ± 0.45	11.43 ± 0.43	11.85 ± 0.51	N.S		−2.22 ± 0.99	0.42 ± 0.48	^*##*^0.006
^#^End recov. VO_2_ (% of VO_2_ Max)	23.52 ± 0.64	21.31 ± 0.68	22.44 ± 0.98	23.27 ± 1.04	N.S		−2.02 ± 0.64	0.83 ± 0.79	0.01
Δ^#^end recov. HR (bpm)	47.25 ± 2	43.08 ± 2.24	40.3 ± 4	40.3 ± 4.7	N.S		−4.17 ± 2.15	0 ± 5.19	N.S

*RPE, relative perceived exertion; BLA, blood lactate; Sys., systolic; Dias., diastolic; BP, blood pressure; AT, Anaerobic threshold; Max, maximal; HR, heart rate; bpm, beats per minute; recov., recovery*.

# *End recovery is the average value of the last 30 s recorded while seated at the end of the test*.

* *Between ATX post and PLA post*.

## *Value calculated with Mann-Whitney U-test, for non-normally distributing variables*.

However, a significant difference was observed between the two groups post supplementation in the blood lactate concentration measured after the VO_2_ Max test. Additionally, a significant reduction was observed in oxygen uptake at the end of recovery between the pre-supplementation and post-supplementation time points in the ATX group compared to the PLA group (Table 1). Supplemental Figure 2 depicts the VO_2_ values during the test by group, before and after supplementation.

Table 2 lists the results from the HTT. The physiological parameters monitored continuously during the test, including HR, Trec, and Tsk displayed no significant difference between the ATX and PLA groups. During the first, un-supplemented HTT, and the second, supplemented HTT, Basal Trec in both groups was below 37°C, and increased by about 1°C. Heart rate began at nearly 80 bpm and increased to just under 120 bpm in both groups.

**Table 2:** Main HTT findings: This table lists the main findings from the HTT performed before (pre) and after (post) supplementation in both treatment groups.

**Test parameter**	**ATX pre**	**ATX post**	**PLA pre**	**PLA post**	**ANOVA/Mann-Whitney U**		**Delta ATX**	**Delta PLA**	***T*-test/Mann-Whitney U**
	**Mean ± St. Error**	**Mean ± St. Error**	**Mean ± St. Error**	**Mean ± St. Error**	***p*-value**	***post hoc* Tukey *p*-value**	**Mean ± St. Error**	**Mean ± St. Error**	***p*-value**
Basal Trec (°C)	36.93 ± 0.08	36.87 ± 0.08	36.95 ± 0.07	36.82 ± 0.12	N.S		−3.13 ± 3.04	−0.12 ± 0.12	N.S
Max Trec (°C)	37.79 ± 0.1	37.9 ± 0.1	37.79 ± 0.1	37.91 ± 0.17	N.S		−3.05 ± 3.12	0.12 ± 0.16	N.S
Δ Trec (°C)	0.83 ± 0.09	0.93 ± 0.12	0.9 ± 0.1	1.1 ± 0.18	N.S		0.1 ± 0.09	0.2 ± 0.2	N.S
End + 1 h Trec (°C)	37.29 ± 0.06	37.26 ± 0.13	37.26 ± 0.07	37.19 ± 0.14	N.S		3.07 ± 3.13	7.38 ± 4.97	N.S
Basal Tskin (°C)	35.41 ± 0.21	35.12 ± 0.24	35.68 ± 0.24	35.49 ± 0.24	N.S		−0.26 ± 0.2	−7.29 ± 4.76	N.S
Max Tskin (°C)	36.4 ± 0.14	36.43 ± 0.23	36.12 ± 0.17	36.28 ± 0.29	N.S		−3 ± 3.01	−7.1 ± 4.83	N.S
Δ Tskin (°C)	0.99 ± 0.22	−2 ± 3.41	0.4 ± 0.27	0.67 ± 0.37	N.S		−2.74 ± 3.07	0.2 ± 0.36	N.S
Sweat rate^*^BSA^−1^	−342.81 ± 23.25	−334.17 ± 22.53	−472.89 ± 29.72	−333 ± 19.71	<0.001	0.002*	8.64 ± 36.11	139.89 ± 39.16	0.023
HR Start (bpm)	79.17 ± 3.29	79.67 ± 3.17	80.4 ± 4.37	79.1 ± 3.84	N.S		0.5 ± 2.91	−1.3 ± 2.78	N.S
HR end (bpm)	118 ± 3.84	116.08 ± 4.71	117.7 ± 6.38	118.9 ± 5.47	N.S		−1.92 ± 4.15	1.2 ± 3.57	N.S
Δ HR (bpm)	38.83 ± 2.29	36.42 ± 5.09	37.3 ± 5.11	39.8 ± 3.68	N.S		−2.42 ± 5.32	2.5 ± 3.42	N.S
RPE start	8 ± 1	7 ± 0	7 ± 0	7 ± 0	N.S		0 ± 1	0 ± 1	N.S
RPE end	11 ± 1	8 ± 0	9 ± 1	10 ± 2	N.S		−1±	0 ± 2	N.S
TCR start	9 ± 1	7 ± 1	8 ± 0	8 ± 1	N.S		−1 ± 1	1 ± 1	N.S
TCR end	11 ± 1	11 ± 1	9 ± 1	9 ± 0	N.S		0±	0 ± 1	N.S
MAP Start (mmHg)	83.78 ± 2.72	89.39 ± 5.03	80.57 ± 9.23	83.73 ± 2.36	N.S		5.61 ± 5.19	3.17 ± 8.96	N.S
MAP end (mmHg)	81.33 ± 2.36	80.36 ± 2.96	83.8 ± 3.69	80.97 ± 2.01	N.S		1.27 ± 2.92	−2.83 ± 4.7	N.S
Δ MAP (mmHg)	−9.22 ± 6.94	−9.03 ± 5.13	3.23 ± 11.59	−2.77 ± 2.4	N.S		0.19 ± 7.92	−6 ± 10.89	N.S
MAP HTT + 1 h (mmHg)	83.48 ± 2.47	90.42 ± 1.23	87.6 ± 4.01	85.17 ± 2.24	N.S		6.67 ± 2.41	−2.43 ± 4.46	N.S

*Max, maximal; Trec, rectal temp.; Tskin, skin temp.; BSA, body surface area; HR, heart rate; bpm, beats per minute; RPE, relative perceived exertion; TCR, thermal comfort rate; MAP, Mean arterial pressure*.

* *Between PLA pre and ATX pre and between PLA pre and PLA post*.

Pre-supplementation sweat rate in the PLA group was significantly higher than the ATX group (which disappeared post-supplementation), and post-supplementation in the PLA group (*p* < 0.001). The subjective scales representing sensations of relative perceived exertion (RPE) and thermal comfort (TCR), which were monitored every 15 min during the test and for 1 h after its completion, also displayed no difference between groups or exposures. Participants perceived a mild to moderate effort in reporting their subjective sensations in the Borg scale (RPE) and moderate heat in the TCR scale.

Biochemical analyses: Table 3 depicts measured serum concentrations of CRP, CPK, HSP72, and the lipid profile, including, high density lipoproteins (HDL), low density lipoproteins LDL total cholesterol and Triglycerides. No significant differences were observed between the ATX and PLA groups in the serum levels of HSP72 protein, in the lipid and triglyceride profile, in CRP or in CPK concentrations, both before and after the effort. However, during all HTT testing days, CPK levels obtained before the test were significantly lower than those obtained immediately after the test, in both groups, both before and after supplementation.

**Table 3:** Serum levels of CPK (mg/Liter), CRP (mg/Liter), HSP72 (ng/mL), and lipid profile: HDl, LDL, total cholesterol and triglycerides (mg/dL).

**Supp**.	**Time point**	**ATX**	**PLA**	***T*-test**\**Mann-Whitney U**
		**Mean ± St. Error**	**Mean ± St. Error**	
CRP pre-supp.	Before HTT	1.09 ± 0.32	0.6 ± 0.14	N.S
	After HTT	1.08 ± 0.33	0.63 ± 0.14	
	After HTT + 1 h	1.06 ± 0.3	0.62 ± 0.13	
	^#^Δ HTT	−0.01 ± 0.03	0.03 ± 0.01	
	^##^Δ HTT + 1 h	−0.03 ± 0.03	0.02 ± 0.02	
CRP post-supp.	Before HTT	1.54 ± 0.46	1.31 ± 0.79	
	After HTT	1.53 ± 0.45	1.22 ± 0.72	
	After HTT + 1 h	1.55 ± 0.46	1.25 ± 0.76	
	^#^Δ HTT	−0.01 ± 0.04	−0.08 ± 0.07	
	^##^Δ HTT + 1 h	0.01 ± 0.07	−0.05 ± 0.03	
CPK pre-supp.	Before HTT	221.42 ± 61.68	188.7 ± 36.69	
	After HTT	260.09 ± 69.54	208 ± 39.68	
	After HTT + 1 h	234.42 ± 57.17	198.8 ± 33.96	
	^#^Δ test	27.64 ± 9.4	19.3 ± 5.06	
	^##^Δ recovery	−15 ± 8.21	−9.2 ± 4.64	
CPK post-supp.	Before HTT	191.5 ± 42.54	134.5 ± 25.83	
	After HTT	207.42 ± 43.94	151.3 ± 26.3	
	After HTT + 1 h	204.75 ± 43.39	146.1 ± 25.9	
	^#^Δ test	15.08 ± 4.7	16.8 ± 3.11	
	^##^Δ recovery	−2.17 ± 3.35	−5.2 ± 2	
HSP72 pre-supp.	Before HTT	1.9 ± 0.75	4.06 ± 0.62	0.021
	After HTT	2.68 ± 0.88	3.77 ± 0.67	N.S
	^**^Δ HTT	0.77 ± 0.54	−0.3 ± 0.53	
HSP72 post-supp.	Before HTT	2.25 ± 0.79	3.75 ± 0.77	
	After HTT	2.31 ± 0.76	3.77 ± 0.77	
	^**^Δ HTT	0.06 ± 0.09	0.03 ± 0.09	
HSP72 ^*^ΔSupp.	Δ before HTT	0.35 ± 0.12	−0.32 ± 0.34	
	Δ after HTT	−0.37 ± 0.59	0.01 ± 0.78	
Total cholesterol	Pre-supp.	139 ± 9.82	156.6 ± 5.98	
	Post-supp.	144.5 ± 7.12	160.5 ± 7.12	
	^*^Δ	5.5 ± 8.14	3.9 ± 5.12	
Triglycerides	Pre-supp.	88.25 ± 16.18	98 ± 11.62	
	Post-supp.	94.33 ± 15.78	95.2 ± 11.62	
	^*^Δ	6.08 ± 8.25	−2.8 ± 9.88	
HDL	Pre-supp.	46 ± 2.7	47.2 ± 2.92	
	Post-supp.	46.42 ± 2.04	47.8 ± 2.12	
	^*^Δ	0.42 ± 1.66	0.6 ± 1.42	
LDL	Pre-supp.	96.17 ± 5.29	105.7 ± 6.29	
	Post-supp.	94.42 ± 5.68	108.8 ± 6.69	
	^*^Δ	−1.75 ± 3.85	3.1 ± 3.74	

*n = 12 in the ATX group, and 10 in the PLA group*.

*HSP72 protein levels were measured in eight participants of each group using ELISA before the HTT and 1 h after completion of the test. Analysis was made in triplicate and averaged for each measurement*.

# *Calculated by subtracting before HTT from after HTT values*.

## *Calculated by subtracting before HTT from after HTT + 1 h values*.

* *Supplementation delta was calculated by subtracting pre supplementation values from the post supplementation values for the appropriate time point*.

** *Delta (Δ) at each HTT was calculated by subtracting Before HTT results from the After HTT results*.

*Supp., supplementation; HTT, heat tolerance test; Δ, delta, HDL, high density lipoproteins; LDL, low density lipoproteins*.

*For CRP, Friedman's omnibus test between time points within treatments revealed no statistically significant difference*.

*For CPK, 1-way ANOVA revealed no significant difference for any of the time points between the ATX and PLA groups. Friedman's omnibus test, followed by Wilcoxon signed-ranks test revealed significant differences between CPK values before and after the HTT (p = 0.005 and p < 0.01 in the ATX and PLA groups, respectively) and in the ATX group, between after the HTT and 1 h after its completion (p = 0.006)*.

*Analysis of HSP72 with Wilcoxon signed ranks test revealed no significant difference between measurements within treatment groups*.

*Analysis of TG's by Wilcoxon signed-ranks test revealed no significant difference between time points within treatment groups*.

*Analysis of Cholesterol and lipids by paired samples t-test revealed no significant difference between measurements within treatment groups*.

## Discussion

We examined the influence of 1 month of 12 mg daily Astaxanthin supplementation on heat tolerance and aerobic capacity. Astaxanthin improved exercise recovery but had no influence on performance in the heat.

Human exercise models, in contrast to animal studies have shown conflicting results regarding the effects of Astaxanthin on performance. For example: the beneficial effects of Astaxanthin in competitive cyclists shown while consuming 4 mg/day (Earnest et al., 2011), vs. no significant difference in performance of well-trained cyclists while consuming 20 mg/day (Res et al., 2013). Neither metabolic markers nor blood biochemistry of human cohorts revealed dose or time dependent metabolic changes attributable to Astaxanthin supplementation (Karppi et al., 2007; Earnest et al., 2011; Res et al., 2013). The variance of substrate oxidation profiles during exercise existing in the general population and the steady state nature of the measurement may have masked a metabolic supplementation effect. The graded VO_2_ Max test used in our study, designed to answer the questions raised regarding the influence of Astaxanthin on substrate utilization in exercising humans over a range of exercise intensities (Brown et al., 2018), showed no effect on aerobic capacity or its components: energy substrate use during the VO_2_ Max test displayed no supplementation effect to influence fat utilization over carbohydrates in either group, as demonstrated in Supplemental Figure 1.

However, the change in blood lactate concentration after the VO_2_ Max test (Table 1), along with the significant reduction in oxygen uptake at the end of recovery in the ATX group compared to the PLA group, may suggest less oxidative stress and faster recovery in comparison with the control, which is a possible advantage for Astaxanthin supplementation. In Supplemental Figure 2, a more rapid return to lower VO_2_ values during recovery is seen in ATX after supplementation compared to before supplementation.

Though evidence from animal models suggests that post exercise recovery may improve with Astaxanthin administration, particularly, by diminishing exercise induced tissue damage markers such as creatine kinase (CK) and myeloperoxidase (MPO), through anti-oxidative and anti-inflammatory pathways (Aoi et al., 2003; Guo et al., 2018), human studies are ambiguous: muscle soreness, exercise force production and plasma CK displayed no significant difference in highly trained individuals who received 3 weeks of 4 mg/day Astaxanthin (Bloomer et al., 2005). However, longer supplementation (90 days) in young soccer players was associated with improved indirect damage markers like reduced lactate dehydrogenase (LDH), and non-significant improvements in CK and inflammatory markers including CRP and leukocyte and neutrophil counts (Djordjevic et al., 2012). Validated information on the effects of Astaxanthin supplementation on exercise performance and recovery, particularly in diverse populations, is lacking.

In the present experiment, though exercise recovery of oxygen uptake was improved in the Astaxanthin group post-supplementation, contrastingly, serum inflammation (CRP), muscle damage (CPK) and lipid profile remained unaffected by supplementation in both groups.

The physiological strain induced by the HTT, was mild for both groups (Trec < 38°C and HR < 120 bpm), as supported by the lack of change in HSP72 post-exercise, pointing to an insufficient perturbation of the thermoregulatory system and the absence of an HSR. Notably, experimental conditions, particularly the physiological safety thresholds, were limited by ethical constraints, and could not induce a higher thermal threshold. Under the experimental conditions employed in this study, no participant reached the safety threshold during heat exposure.

An Additional component contributing to the observed physiological response may have been the fitness level of participants and the relatively mild effort undertaken by them during the HTT. An average VO_2_ Max of 51–52 ml × kg^−1^ × min^−1^ was typical of the study participants. The average HR elevation during the HTT was ~40 bpm, reflecting a 21% change relative to the measured maximal HR in the VO_2_ Max test (Table 1), indicating a state of mild stress experienced during the HTT across treatment groups and exposures.

Nevertheless, the significant difference in CPK levels from the beginning to the end of the HTT, in both groups indicates some muscle damage resulting from the HTT, which was unaffected by supplementation (Table 3).

The significantly higher sweat rate in the pre-supplemented PLA group compared to PLA post-supplementation and to ATX pre- and post-supplementation cannot be explained by an effect of supplementation, and can only be attributed to a difference between participant groups. This was, however, insignificant when the change in sweat rate from pre- to post-supplementation was compared between treatment groups (Table 2).

The daily dose of Astaxanthin used in this work (12 mg) was reflective of the highest recommended dose for humans at the time, which has been substantially increased since then to 24 mg daily (Visioli and Artaria, 2017). Consumption of a larger dose may have evoked greater effects in aerobic function and cellular protective aspects important to coping with the damages of heat stress exposure.

## Conclusion

Preemptive nutritional supplementation is a promising avenue for exercise science research as a way of improving physiological resilience in preparation for an anticipated exposure to adverse conditions and to strenuous efforts. Long term supplementation of 12 mg\daily Astaxanthin contributed to improved aerobic recovery, but was not beneficially manifested under the examined heat load conditions. It remains to be seen if administration of larger doses of Astaxanthin or exposure to greater environmental and physiological stress that elicit a heat shock response might bring additional protective mechanisms of Astaxanthin supplementation into light.

## Data Availability

The raw data supporting the conclusions of this manuscript will be made available by the authors, without undue reservation, to any qualified researcher.

## Author Contributions

CF contributed to the conception and design of the study, conducted the experiments, analyzed the data, and wrote the manuscript. MH contributed to data analysis and to manuscript design and reviewed the manuscript. RY contributed to conducting the experiments, to data analysis, and reviewed the manuscript. HR participated as the study nutritionist and contributed to conducting the experiments, to data analysis, and reviewed the manuscript. YH contributed to the conception and design of the study, to conducting the experiments, and reviewed the manuscript.

### Conflict of Interest Statement

The authors declare that the research was conducted in the absence of any commercial or financial relationships that could be construed as a potential conflict of interest.

## Supplementary Material

Supplemental Figure S1 Scatter plot of RER vs. VO_2_ during the VO_2_ Max tests, for the ATX and PLA supplementation groups. The ATX group before and after supplementation is represented by the blue and gray dots, respectively. The PLA group before and after supplementation is represented by the orange and yellow dots, respectively.

Supplemental Figure S2 VO_2_ Max test. Depicts the VO_2_ Max test graphs by group and stage: **(A)** upper left, green lines: ATX group, before supplementation; **(B)** lower left, brown lines: ATX group, after supplementation; **(C)** upper right, blue lines: PLA group, before supplementation; **(D)** lower right, black lines: PLA group, after supplementation.

## Abbreviations

AT: Anaerobic threshold
ATX: Astaxanthin
BMI: Body mass index
BSA: Body surface area
CPET: Cardio pulmonary exercise test
CPK: Creatine phospho kinase
CRP: C- reactive protein
ELISA: Enzyme-Linked Immunosorbent Assay
FDA: U.S. Food and Drug Administration
GI: Gastro intestinal
HR: Heart rate
HSP72: Heat shock protein 72
HSR: Heat shock response
HTT: Heat tolerance test
IGF-1: Insulin-like growth factor 1
MAP: Mean arterial pressure
MPO: Myeloperoxidase
MSK-1: Mitogen- and stress-activated protein kinase-1
OBLA: Onset of blood lactate accumulation
OD: Optical density
PLA: Placebo
PP: Pulse pressure
RER: Respiratory exchange ratio
RH: relative humidity
RONS: Reactive oxygen and nitrogen species
ROS: Reactive oxygen species
RPE: Relative perceived exertion
SR: Sweat rate
TCR: Thermal comfort rate
VO_2_: Oxygen uptake
VO_2_ Max: Maximal oxygen uptake.
