# Supplementary figures and images for: Asthaxanthin Improves Aerobic Exercise Recovery Without Affecting Heat Tolerance in Humans
Source: Front Sports Act Living. 2019 Sep 4;1:17. doi: 10.3389/fspor.2019.00017 (PMC7739736; doi:10.3389/fspor.2019.00017)

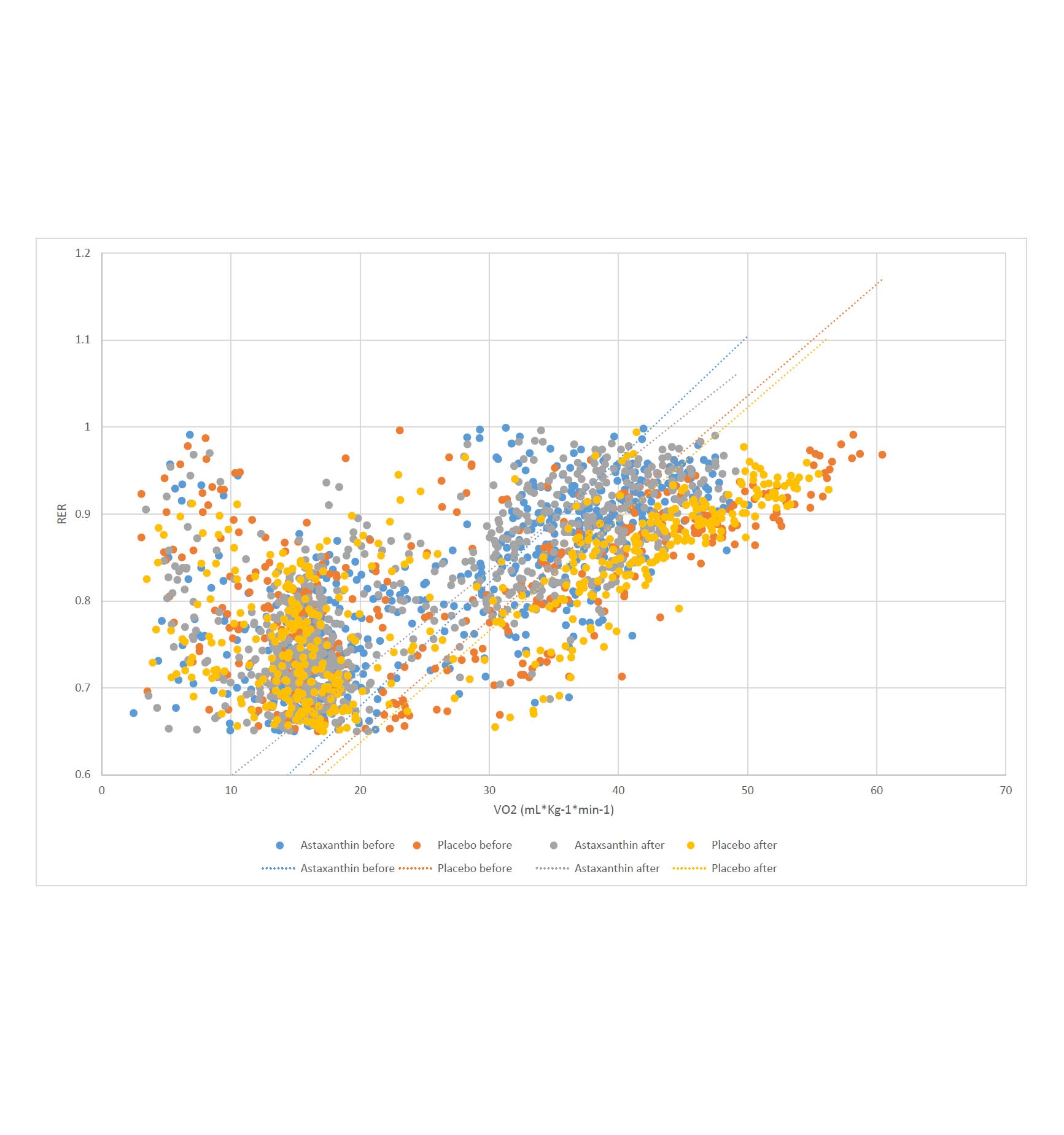

Supplement: Supplemental Figure S1 — Scatter plot of RER vs. VO2 during the VO2 Max tests, for the ATX and PLA supplementation groups. The ATX group before and after supplementation is represented by the blue and gray dots, respectively. The PLA group before and after supplementation is represented by the orange and yellow dots, respectively. [file Image_1.JPEG]

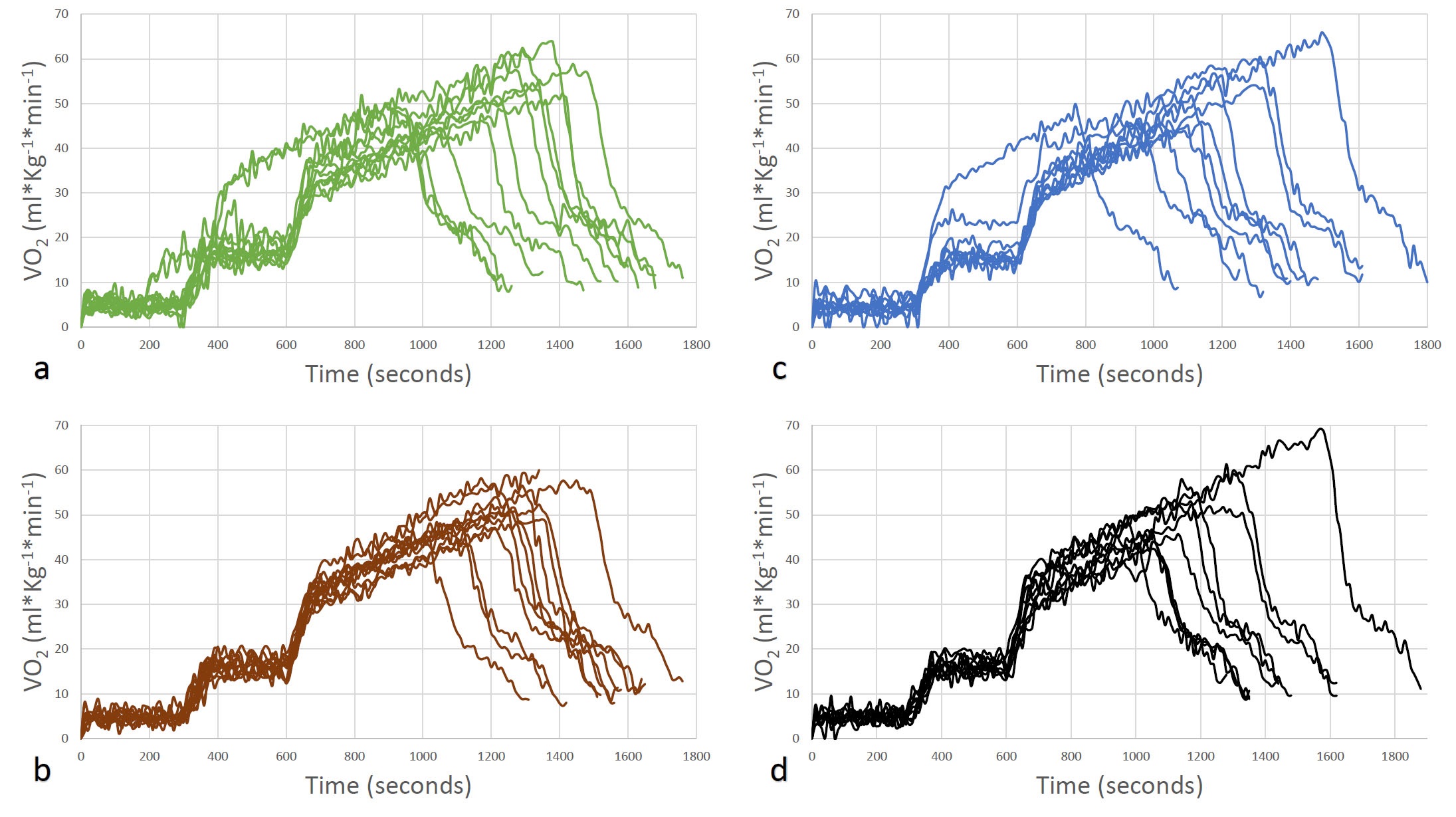

Supplement: Supplemental Figure S2 — VO2 Max test. Depicts the VO2 Max test graphs by group and stage: (A) upper left, green lines: ATX group, before supplementation; (B) lower left, brown lines: ATX group, after supplementation; (C) upper right, blue lines: PLA group, before supplementation; (D) lower right, black lines: PLA group, after supplementation. [file Image_2.JPEG]
